# Algorithmic Foundation of Spectral Rarefaction for Measuring Satellite Imagery Heterogeneity at Multiple Spatial Scales

**DOI:** 10.3390/s90100303

**Published:** 2009-01-08

**Authors:** Duccio Rocchini

**Affiliations:** 1 Dipartimento di Scienze Ambientali “G. Sarfatti”, Università di Siena, via P.A. Mattioli 4, 53100 Siena, Italy; E-mail: rocchini@unisi.it; 2 TerraData *environmetrics*, Università di Siena, via P.A. Mattioli 4, 53100 Siena, Italy

**Keywords:** algorithmic solution of rarefaction, rarefaction theory, satellite imagery, spectral heterogeneity

## Abstract

Measuring heterogeneity in satellite imagery is an important task to deal with. Most measures of spectral diversity have been based on Shannon Information theory. However, this approach does not inherently address different scales, ranging from local (hereafter referred to alpha diversity) to global scales (gamma diversity). The aim of this paper is to propose a method for measuring spectral heterogeneity at multiple scales based on rarefaction curves. An algorithmic solution of rarefaction applied to image pixel values (Digital Numbers, DNs) is provided and discussed.

## 1. Introduction

Measuring heterogeneity in satellite imagery is important, since heterogeneity in an image represents the degree of diversity of objects reflecting within a landscape. In fact, since the IFOV (Instantaneous Field of View) of an image represents a spatially implicit representation of reality, each pixel is expected to represent reality at a certain resolution.

Despite the attribute being considered, the diversity of that attribute has been proven to change as a function of scale [1]. Most measures of spectral diversity have been proposed based on the Boltzmann index [2-3], commonly referred to as Shannon entropy index [4-6] *H* = −Σ*p* × ln(*p*), where *p* is the relative abundance of each spectral reflectance value (Digital Number, DN). The Shannon index will increase if the DN values are equally distributed with no DN value being dominant with respect to the others. The Shannon index has been advocated as a powerful algorithm for measuring diversity. Nonetheless, it does not explicitly consider how the measure of diversity changes as a function of scale if it is applied to an entire image. It may be made to account for the variation of diversity across spatial scales if it is repeatedly calculated while increasing the sampling extent within the chosen study area. This process may be time expensive. Quoting Gorelick [3], who made a critique on diversity measured by Shannon and Simpson indices, one can never capture all aspects of diversity in a single statistic. This is true regardless of the attribute being considered.

The aim of this paper is to propose a method for measuring spectral heterogeneity at multiple scales simultaneously based on ecological theory.

## 2. Algorithmic foundation of spectral rarefaction

In ecology, there is a long history of dealing with species diversity over space or time. In particular, given *N* plots, i.e. sampling units with a certain dimension, three different kinds of species diversity may be recognized:

- alpha or local diversity (α), i.e. the number of species within one plot
- gamma or total diversity (γ), i.e. the number of species considering *N* plots
- beta or between-plots diversity (β), i.e. the diversity deriving from the complementarity of the species composition considering pairs of plots [1].

In this view, accumulation curves, showing the number of accumulated species given a certain number of sampled plots, have long been used for estimating the expected number of species within a study area given a specific sampling effort. Since the order that samples are added to an accumulation curve accounts for its shape [7-8], an order-free curve is derived by means of (i) an analytical solution or of (ii) permutations of samples [9-10]. This order-free curve is referred to as a rarefaction curve. Considering permutation (ii), once *N* plots have been visited across a study area and the presence of all species has been recorded (obtaining a presence/absence matrix **M_s_** of *N* plots per *S* species), a rarefaction curve is then obtained by repeatedly resampling the pool of *N* plots at random without replacement and plotting the average number of species represented by *1, 2, …, N* plots [6,10]. Thus, sample-based rarefaction generates the expected number of accumulated species as the number of sampled plots increases from 1 to *N*.

On the other hand, an analytical solution (i) may be formalized as:

Let **M_s_** be a presence/absence matrix of *N* plots per *S* species, the formal estimate of the expected number of species per number of plots turns out to be:

(1) $$E[S]=S-\frac{\overset{S}{\underset{i=1}{\text{∑}}}\left(\begin{array}{l} N-N_{i}\\ n \end{array}\right)}{\left(\begin{array}{l} N\\ n \end{array}\right)}$$

where *N_i_* = number of plots where species *i* is found and *n* = number of randomly chosen plots [9-12]

Generally, the steeper the curve, the greater the increase in species richness as the sample size increases [7-12].

From a landscape perspective, rarefaction curves are directly related to the environmental heterogeneity of the area sampled. In fact, it is expected that the greater the landscape heterogeneity, the greater the species diversity, including both fine-scale and coarse-scale species richness (i.e. α- and γ-diversity, respectively), and compositional variability, or β-diversity [7].

Computing β-diversity deals with looking at the difference between pairs of plots in terms of species composition [13-15]. Popular indices of β-diversity, e.g. the Jaccard index, are based on the intersection of the composition in species between pairs of plots with respect to their union, as *C_j_* = 1 − (∩/∪) [13]. The higher the intersection in species composition the lower the β-diversity. As an example, given two plots with α = 5 species, using *C_j_*, β-diversity will range from β=0 when the 5 species will be exactly the same, while the maximum β-diversity (β=1) will occur when all the 5 species will be different.

**Figure 1.**
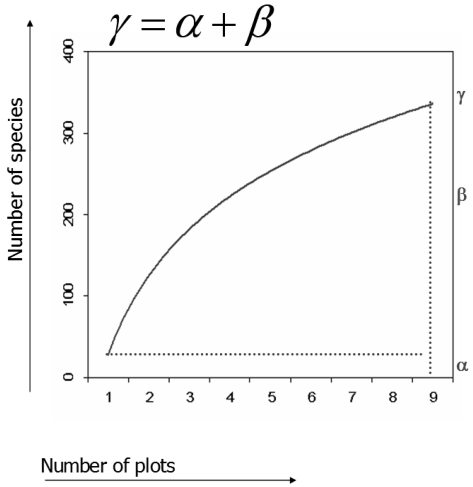
Additive partitioning of diversity. γ-diversity is represented by the sum between α and β. This leads to consider β in the same unit of measurement (i.e. number of species) of α and γ.

An alternative definition of β-diversity has been provided by Whittaker [1] who expressed it as *β* = *γ/ᾱ*. This was later modified by Lande [16] being more consistent with the rarefaction theory. Using rarefaction curves, diversity may be partitioned by additive partitioning as *γ* = *α* + *β* [16-17], leading to considering β in the same unit of measurement (i.e. number of species) of α and γ as: *β* = *γ* − *α* (Fig 1).

In this paper, different “species” will be replaced by different “DNs” (Digital Numbers, i.e. spectral values).

Consider a satellite image with a radiometric resolution of 8 bit. This means that the reflectance values of the pixels, i.e. the Digital Numbers (DNs), may range from 0 to 255.

Subsampling the image by means of *N* plots, i.e. spatial windows with a certain dimension, will lead to a presence/absence matrix **M_DN_** of *N* plots per *S* DNs.

Given the matrix **M_DN_**, Eq.(1) previously introduced for species diversity can also provide a formal estimate of the number of DNs per number of windows when *N_i_* = number of plots where the DN value *i* is found.

Therefore, the same concepts introduced for species diversity may thus be applied to satellite imagery diversity. Applying rarefaction theory to DNs rather than species leads to consider three different components of pixels diversity:

- alpha or local diversity (α_DN_), i.e. the number of different DNs within one plot
- gamma or total diversity (γ_DN_), i.e. the number of different DNs considering *N* plots
- beta or between-plots diversity (β_DN_), i.e. the diversity deriving from *β*_DN_ = *γ*_DN_ − *α*_DN_ [16-17].

## 3. Worked example

Eq.(1) only works with one-dimensional systems. In fact the dimension Dim(**M_DN_**) of the presence absence matrix **M_DN_** of *N* plots per *S* DN values equals Dim(**M_DN_**)=(*N,S*), implying that:

- the plots are rows
- the DN values are columns
- the cells composing the matrix are presence/absence values, i.e. they are dummy coded as 1s and 0s.

**Figure 2.**
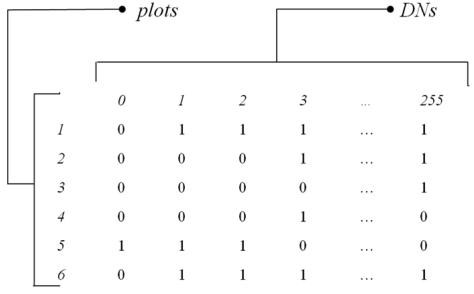
The presence/absence matrix **M_DN_** of *N* plots per *S* DN values. Notice that only one band can be considered at once, with DN values in one dimension ranging from 0 to 255.

For instance, Fig. 2 shows the presence/absence matrix **M_DN_** of *N* plots per *S* DN values derived from an 8-bit image sampled by 6 plots, where Dim(**M_DN_**)=(*N,S*)=(6,256), with DN values in one dimension ranging from 0 to 255.

Thus, before building rarefaction curves one should choose a single band to work with. Following biological theory, an infrared waveband should be used when working with vegetation based on its intrinsic capability of discriminating different vegetation types [18]. Another option may be based on performing data reduction with a method such as PCA and further using the first principal component explaining most of the variance.

**Figure 3.**
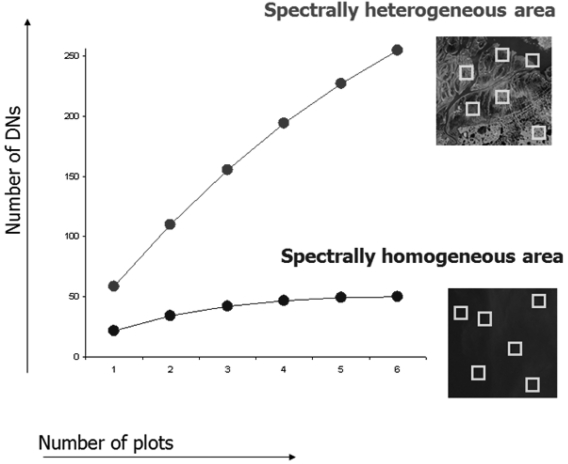
A worked example of spectral rarefaction.. Once differently heterogeneous areas are sampled by the same number of plots (windows) containing the same number of inner pixels, the rarefaction curves computed by Eq.(1) provide an estimate of the number of different DNs at various spatial scales. Obviously only one band or the first PC can be used at once. See the main text for major explanations.

Once the rarefaction algorithm (Eq.(1)) has been applied to the presence absence matrix **M_DN_**, different study areas sampled by the same number of plots containing the same number of inner pixels (e.g. 1000 pixels per spatial window) will possibly show very different curves. Fig. 3 shows two areas with different levels of heterogeneity, each sampled by six spatial windows (plots).

Considering the ecologically heterogeneous area (upper curve of Fig. 3) with respect to the more homogeneous one (lower curve of Fig. 3), α_DN_ equals 55 and 24 respectively, i.e. there are on average 55 and 24 distinct reflectance values for each plot (spatial window).

Meanwhile γ turns out to be 253 and 50, for heterogeneous vs. homogeneous area, respectively.

This means that the spectral value diversity β_DN_ as calculated by γ_DN_-α_DN_ is 198 and 26, respectively.

Notice that in this worked example, the rarefaction algorithm (Eq.(1)) allowed us to: (i) represent the variation in diversity by means of a single algorithm applied to a matrix, i.e. the spatial variation of diversity components, (ii) represent β-diversity by means of only one statistic without considering pair-wise distances among spatial windows (as in the case of e.g. the Jaccard index), and (iii) represent β-diversity in the same units as α- and γ- diversity (i.e. number of different DN values).

## 4. Remarks and summary

Rarefaction and additive partitioning of diversity, which are often used in ecology with reference to species diversity [6-12,16-17], could be applied to reflectance values for estimating and graphically representing local (α), global (γ) and turnover in (β) environmental variability. In fact, once rarefaction curves are graphed, it becomes apparent that α, γ and β represent the minimum, the maximum and the turnover (i.e. maximum - minimum) of the curve (Fig. 1). Generally speaking, it is expected that the higher the minimum value the higher the local variability within a plot, while, given the same local variability, the higher the slope of the curve the higher the variability across the different plots within the area [7,10,19]. On the contrary if the slope is low, i.e. when the curve rapidly reaches the asymptote, the accumulated spectral values are simply a replicate of the sampled spectral values, thus indicating global homogeneity of the area.

In summary, for each plot (spatial window), containing a number *n* of pixels (e.g. 1000 pixels per window), the number of different DNs should theoretically range from 1 (homogeneous environment such as water) to 256 (heterogeneous environment composed of different land cover classes, with a 8-bit image). Notice that a lower maximum number of DNs per plot (window) is expected on the strength of the spatial autocorrelation of spectral values. Once spectral rarefaction curves are built, the number of DN values per window is directly estimated (α) and rises until theoretically reaching the maximum value of 256 (in case of commonly used 8-bit images) as new plots are added to the curve. Obviously the theoretical maximum of 256 different values is reached only when the considered area is so heterogeneous that it comprises all the 256 values.

The approach proposed for measuring spectral heterogeneity is robust but straightforward and consists of three main tasks: (i) selecting within the image adjacent or random windows containing a given number of pixels; (ii) choosing one band (Eq.(1) only works with one-dimensional systems, see section “2. Worked example”), (iii) performing rarefaction curves by Eq.(1) and estimating α-, β− and γ- diversity components.

Of course, other techniques rather than spectral rarefaction could account for the spatial variability of DN values as well, e.g. semivariograms [14,20]. However, spectral rarefaction coupled with additive partitioning exhibits mathematical and statistical properties which may be directly related to spectral and species α-, β- and γ- diversity. This is an enormous advantage to using spectral rarefaction as a straightforward method for (i) robustly estimating local to global diversity of an area directly relating sensor-based and field-based heterogeneity and (ii) quantitatively comparing different areas with different degrees of heterogeneity at multiple scales.
